# Penetrating Wounds

**Published:** 1892-06

**Authors:** 


PENETRATING ABDOMINAL WOUNDS.—In our "Selections" will be found a very excellent article from *The Epitome* by Dr. W. B. Coley. Mention is made of it here to emphasize the importance of an early operation, if at all. So soon as the primary shock can be overcome, the case should be well considered in all its details, and if an operation is decided on, perform it at once, or not at all. Several operations have been made in this city during the past few years with a certainty of the failure that followed. The idea of waiting twenty-four to forty-eight hours, or in some instances, even longer, as has been done, may assist in perfecting the manual dexterity of the surgeon, but as for giving a chance to the patient, it only insures the chances for his early need of an undertaker.—*Southern Practitioner.*

